# Reproducibility, Performance, and Clinical Utility of a Genetic Risk Prediction Model for Prostate Cancer in Japanese

**DOI:** 10.1371/journal.pone.0046454

**Published:** 2012-10-10

**Authors:** Shusuke Akamatsu, Atsushi Takahashi, Ryo Takata, Michiaki Kubo, Takahiro Inoue, Takashi Morizono, Tatsuhiko Tsunoda, Naoyuki Kamatani, Christopher A. Haiman, Peggy Wan, Gary K. Chen, Loic Le Marchand, Laurence N. Kolonel, Brian E. Henderson, Tomoaki Fujioka, Tomonori Habuchi, Yusuke Nakamura, Osamu Ogawa, Hidewaki Nakagawa

**Affiliations:** 1 Laboratory for Biomarker Development, Center for Genomic Medicine, RIKEN, Tokyo, Japan; 2 Department of Urology, Graduate School of Medicine, Kyoto University, Kyoto, Japan; 3 Laboratory for Statistical Analysis, Center for Genomic Medicine, RIKEN, Tokyo, Japan; 4 Department of Urology, Iwate Medical University, Morioka, Japan; 5 Laboratory for Genotyping Development, Center for Genomic Medicine, RIKEN, Yokohama, Japan; 6 Laboratory for Medical Informatics, Center for Genomic Medicine, RIKEN, Yokohama, Japan; 7 Department of Preventive Medicine, Keck School of Medicine, University of Southern California, Los Angeles, California, United States of America; 8 Epidemiology Program, Cancer Research Centre, University of Hawaii, Honolulu, Hawaii, United States of America; 9 Department of Urology, Akita University School of Medicine, Akita, Japan; 10 Laboratory of Molecular Medicine, Human Genome Center, Institute of Medical Science, the University of Tokyo, Tokyo, Japan; The Chinese University of Hong Kong, Hong Kong

## Abstract

Prostate specific antigen (PSA) is widely used as a diagnostic biomarker for prostate cancer (PC). However, due to its low predictive performance, many patients without PC suffer from the harms of unnecessary prostate needle biopsies. The present study aims to evaluate the reproducibility and performance of a genetic risk prediction model in Japanese and estimate its utility as a diagnostic biomarker in a clinical scenario. We created a logistic regression model incorporating 16 SNPs that were significantly associated with PC in a genome-wide association study of Japanese population using 689 cases and 749 male controls. The model was validated by two independent sets of Japanese samples comprising 3,294 cases and 6,281 male controls. The areas under curve (AUC) of the model were 0.679, 0.655, and 0.661 for the samples used to create the model and those used for validation. The AUCs were not significantly altered in samples with PSA 1–10 ng/ml. 24.2% and 9.7% of the patients had odds ratio <0.5 (low risk) or >2 (high risk) in the model. Assuming the overall positive rate of prostate needle biopsies to be 20%, the positive biopsy rates were 10.7% and 42.4% for the low and high genetic risk groups respectively. Our genetic risk prediction model for PC was highly reproducible, and its predictive performance was not influenced by PSA. The model could have a potential to affect clinical decision when it is applied to patients with gray-zone PSA, which should be confirmed in future clinical studies.

## Introduction

Prostate cancer (PC) is the most frequent non-cutaneous malignancy in the Western countries [1], and its incidence has also been rapidly increasing in Asian countries including Japan [2]. Although environmental factors such as fat-rich or high-calorie diet intake may play some important roles in PC carcinogenesis, the only definitive risk factors for PC are age, family history, and ethnicity [3], [4]. Prostate specific antigen (PSA) is widely used as a diagnostic biomarker for PC. Patients with PSA above the cutoff value of 2–4 ng/ml are suspected to have PC and undergo invasive transrectal or transperineal prostate needle biopsies. However, the predictive performance of PSA is recognized to be unfavorable, especially for patients with ‘gray-zone’ PSA of less than 10 ng/ml. Only 20–25% of the patients with gray-zone PSA are histologically diagnosed with PC at biopsy [5], [6], [7]. Hence, many patients without PC suffer from the harms of unnecessary prostate biopsies such as pain, hematuria, rectal bleeding, prostatitis, and sepsis [8]. The usefulness of compensatory indexes such as PSA velocity, PSA density and free to total PSA ratio are limited due to their dependence on PSA [5], [9], and performances of other biomarkers such as PCA3 are still under investigation [10]. Therefore, novel biomarkers that can risk stratify patients at gray-zone PSA to decide who should be recommended to undergo prostate biopsies are urgently needed.

Recently, more than forty genomic loci associated with susceptibility to PC have been identified by genome-wide association studies (GWAS) [11], [12], [13], [14], [15], [16], [17], [18], [19], [20]. Besides identifying new genes or pathways to clarify the etiology of diseases, information gained from GWAS can also be utilized to estimate predispositions to developing diseases [21]. However, the effect size, or odds ratio (OR) of common susceptibility variants identified by GWAS are generally small (1.1–2.0), and the utility of each variant in estimating disease susceptibility is limited. Therefore, efforts have been made to develop genetic risk prediction models incorporating multiple susceptibility variants [22].

We have previously identified five novel loci associated with PC in the GWAS of Japanese, and also reported that although 19 of the 31 then reported loci were replicated (*P-value*<0.05) in the Japanese, 12 were not, confirming the presence of ethnic heterogeneity in genetic susceptibility to PC [20]. Some of the five novel loci we have identified were later replicated in a Caucasian population at lower odds ratios and some were not [23], [24]. These data suggest that genetic risk prediction models should be individualized to each ethnic group. In the present study, we have created a genetic risk prediction model for PC based on 16 genetic variants that were associated with PC in the GWAS of Japanese, and confirmed its reproducibility in two independent sets of Japanese samples. We have also tested the performance of the model in a clinical scenario and estimated whether the model could be clinically and practically useful to risk-stratify patients at gray-zone PSA.

## Materials and Methods

### Genetic Variant selection and creation of a genetic risk prediction model

Of the 31 PC susceptibility germline variants reported by July 2010, 15 variants passed the threshold of significance after Bonferroni correction (*P*-value<1.6×10^−3^) in our GWAS stage 1, including multiple variants at *8q24* (**Table S1**) [20]. There are five independent regions associated with PC in *8q24* [25]. The variants that showed the strongest association in the Japanese GWAS for each of the five regions were selected to be included in the model. We have previously fine-mapped so-called Region 2 of *8q24* [26], and rs1456315, which showed the strongest association with PC (*P* = 2.00×10^−24^, OR = 1.74) in the Japanese in this region was chosen for Region 2 of *8q24*. Five novel variants we have identified in the Japanese GWAS [20] were also included, and the total of 16 variants were incorporated into a genetic risk prediction model for PC as explanatory variables in a logistic regression model (**Table 1**).No variants were within the same linkage disequilibrium block, and all the variants selected were not correlated with (r^2^<0.2) each other. For creation of a risk prediction model, each sample was scored for each of the 16 variants with the number of risk alleles (0, 1, and 2). A genetic risk prediction model was created by unconditional logistic regression incorporating all 16 SNPs. Odds ratio (OR) was estimated for each sample based on the model from the following formula:
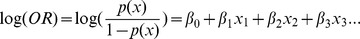
where 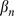 are the regression coefficients of the each SNP and 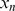 are the number of the risk alleles at the each SNP locus. We also evaluated our genetic prediction model by two independent Japanese sample sets not used for the model construction.

**Table 1:** 16 SNPs incorporated in the risk prediction model and their regression coefficients in the model.

Chr	Region	ref SNP ID	*P*-value^a^	Odds ratio (95% CI)^b^	Regression coefficient (β)^c^
2	*C2orf43*	rs13385191	5.32E-06	1.22 (1.12–1.33)	0.040
2	*THADA*	rs11693801	6.88E-06	1.23 (1.13–1.35)	0.118
3	*3p12*	rs9284813	5.10E-09	1.34 (1.22–1.48)	0.243
5	*IRX4*	rs12653946	8.31E-10	1.32 (1.20–1.43)	0.214
6	*FOXP4*	rs1983891	2.03E-06	1.23 (1.13–1.34)	0.166
6	*RFX6/GPRC6A*	rs339331	6.00E-08	1.28 (1.17–1.40)	0.236
8	*NKX3.1*	rs1512268	4.25E-11	1.34 (1.23–1.46)	0.136
8	*8q24 (Block1)*	rs10086908	6.94E-06	1.29 (1.15–1.43)	0.152
8	*8q24 (Block2/Region2)*	rs1456315	1.62E-29	1.75 (1.59–1.93)	0.475
8	*8q24 (Block3/Region3)*	rs620861	7.17E-04	1.16 (1.06–1.26)	−0.011
8	*8q24 (Block4/Region3)*	rs6983267	2.89E-06	1.23 (1.13–1.34)	0.268
8	*8q24 (Block5/Region1)*	rs7837688	1.21E-25	1.86 (1.59–1.96)	0.644
10	*MSMB*	rs10993994	3.44E-08	1.27 (1.17–1.38)	0.238
13	*13q22*	rs9600079	7.71E-05	1.19 (1.09–1.30)	0.133
17	*HNF1B*	rs7501939	1.24E-12	1.41 (1.28–1.54)	0.313
22	*TTLL1/BIK*	rs5759167	5.77E-04	1.17 (1.07–1.29)	0.127
					Intercept d = −3.511

a *P* for trend (1-degree of freedom) in GWAS stage 1.

b Odds ratio and confidence intervals of risk alleles in multiplicative models in GWAS stage 1.

c Regression coefficients of each SNP in the risk prediction model.

d Intercept of the risk prediction model.

### Samples and genotyping

The basic characteristics of the study participants are summarized in **Table 2**. The samples that were used to create the model (*AKY*) were drawn from Akita-Kyoto Cohort (*AKYC*) including 732 cases and 957 controls [27]. The case samples were recruited from Japanese PC patients at Kyoto University and Akita University. All case samples were histologically diagnosed by local pathologists, and all clinical data were collected by local urologists. The control samples of *AKYC* included Japanese samples collected from the male patients visiting the urology clinics at the two university hospitals with diseases other than PC and other malignancies, and from healthy male volunteers visiting for medical checkups. The control patients had at least one PSA tests every 1–2 years along with digital rectal exams. Although the exact PSA data for each sample is missing, patients with PSA>4 ng/mL were not included in the current study controls except those who received prostate needle biopsy along with surgery due to benign prostatic hyperplasia, and had no malignancy in the pathological specimen. *AKYC* samples were genotyped by multiplex PCR-based invader assays [28]. Sample call rates were >99%, and there was no deviation from Hardy-Weinberg equilibrium for all the 16 SNPs among the controls. After genotyping, 689 cases with full genotype and age data and 749 controls that were matched by age groups of 5-year intervals were drawn to create the genetic risk prediction model. The samples used for the 1^st^ validation study (*BBJ*) were drawn from stage 2 of our GWAS of Japanese [20]. All PC cases and male controls were obtained from BioBank Japan at the Institute of Medical Science, the University of Tokyo [29]. This project was started in 2003 to collect a total of 300,000 cases that have at least one of the 47 diseases by a collaborative network of 66 hospitals located at all areas of Japan. Of the 3,001 case and 5,415 male control samples used for the replication study in the GWAS, full genotype data was available for the 16 SNPs in 2,950 cases and 5,235 controls. The 2^nd^ validation study (*BBJ2*) is a nested case-control study using sample drawn from BioBank Japan. The 344 cases of *BBJ2* had been enrolled in BioBank Japan as patients with diseases other than PC during 2003–2007 and were subsequently diagnosed with PC during follow-up periods (at most 7 years). The 1,045 controls of *BBJ2* were drawn from 14,541 male samples used in GWAS for 18 diseases other than PC. The controls were matched by age groups of 5-year intervals and by the follow-up years with the cases: control ratio of 1∶3. The BBJ2 case samples were genotyped for the 16 SNPs by multiplex PCR-based invader assays. The *BBJ2* control samples were genotyped using Illumina HumanHap550v3 or Human 610-Quad Bead Chip. For the *BBJ2* case and control samples, baseline-PSA levels were measured using their sera pooled at the time of the registration to BioBank Japan by chemiluminescent enzyme immunoassay (CLEIA).

**Table 2:** Clinical characteristics of the cases and the controls.

		AKY (Discovery)		BBJ (Validation 1)		BBJ2 (Validation 2)	
			(%)		(%)		(%)
**Controls**							
	Number of samples	749		5236		1045	
	Mean age [s.d.]	68.6	[7.5]	68.4	[10.3]	70.8	[6.8]
	Serum PSA level	N/A		N/A			
	PSA≤1					433	(41.4)
	PSA1–10					575	(55.0)
	PSA≥10					37	( 3.5 )
**Cases**							
	Number of samples	689		2950		344	
	Mean age [s.d.]	67.8	[7.1]	74.0	[7.0]	71.3	[6.7]
	Serum PSA level						
	PSA≤10	362	(52.5)	513	(17.4)	211	(61.3)
	PSA10–20	150	(21.8)	271	(9.2)	60	(17.4)
	PSA≥20	155	(22.5)	400	(13.6)	73	(21.2)
	Missing data	22	(3.2)	1766	(59.9)	0	(0.0)
	Tumor stage					N/A	
	T0	7	(1.0)	6	(0.2)		
	T1	226	(32.8)	224	(7.6)		
	T2	225	(32.7)	345	(11.7)		
	T3	133	(19.3)	200	(6.8)		
	T4	14	(2.0)	32	(1.1)		
	Missing data	84	(12.2)	2143	(72.6)		
	Nodal stage					N/A	
	N0	590	(85.6)	742	(25.2)		
	N1	25	(3.6)	30	(1.0)		
	Missing data	74	(10.7)	2178	(73.8)		
	Metastasis data					N/A	
	M0	585	(84.9)	727	(24.6)		
	M1	56	(8.1)	43	(1.5)		
	Missing data	48	(7.0)	2180	(73.9)		
	Clinical stage			N/A		N/A	
	A	3	(0.4)				
	B	458	(66.5)				
	C	120	(17.4)				
	D	95	(13.8)				
	Missing data	13	(1.9)				
	Gleason score					N/A	
	GS≤6	138	(20.0)	755	(25.6)		
	GS7	238	(34.5)	622	(21.0)		
	GS≥8	152	(22.1)	114	(3.9)		
	Missing data	161	(23.4)	1459	(49.5)		
	High risk PC*	302	(43.8)	N/A		N/A	
	Non-high risk PC	280	(40.6)				
	Missing data	107	(15.5)				

AKY: Akita-Kyoto cohort, BBJ: Biobank Japan cohort, BBJ2: Biobank Japan cohort 2.

* High risk PC fulfills either of the following criteria; PSA≥20, or Gleason Score≥8, or clinical stage≥C.

### Ethics Statement

The research project and the sample collection were approved by the ethical committees in the Institute of Medical Science, the University of Tokyo, Yokohama Institute of RIKEN, Kyoto University, Akita University, University of Southern California, and University of Hawaii. Written informed consent was obtained from all individuals.

### Receiver operating characteristic (ROC) analysis

We used R statistical environment version 2.61 for analysis. R package Epi and pROC were used to draw ROC plots and calculate areas under curve (AUCs). AUCs were statistically compared by paired or unpaired DeLong's test.

### Calculation of positive prostate needle biopsy rates at given cutoffs

Assuming the positive prostate needle biopsy rate before genetic risk prediction to be 20% [5], [6], [7], positive prostate biopsy rate for the risk population, or positive predictive value is (0.2 SE)/{0.2 SE+0.8 (1-SP)} and positive prostate biopsy rate for the non-risk population, or (1-negative predictive value) is 0.2 (1-SE)/{0.2 (1-SE)+0.8 SP}, where SE and SP are sensitivity and specificity of the genetic prediction model at an arbitrary cutoff value.

## Results

### Reproducibility and discriminatory potential of the genetic risk prediction model

The regression coefficients of the risk prediction model were positive for all the 16 variants except rs620861, indicating that the risk alleles of the variants selected based on stage1 of our GWAS are positive risk factors in *AKY* samples except at rs620861 (**Table 1**). rs620861 is in the so-called Block 3 of *8q24*, and was first reported as a PC-susceptibility locus in a GWAS of European ancestry, and was confirmed to be associated with PC in stage 1 of our GWAS of the Japanese. Although this variant showed negative association with PC in *AKY* samples, the association was mildly positive in *BBJ* and *BBJ2* as well as in the meta-analysis of the three sample sets used in the present study (**Table S2**), indicating that the negative association of this variant with PC risk in *AKY* samples may be due to sample collection bias.

**Figure 1:**
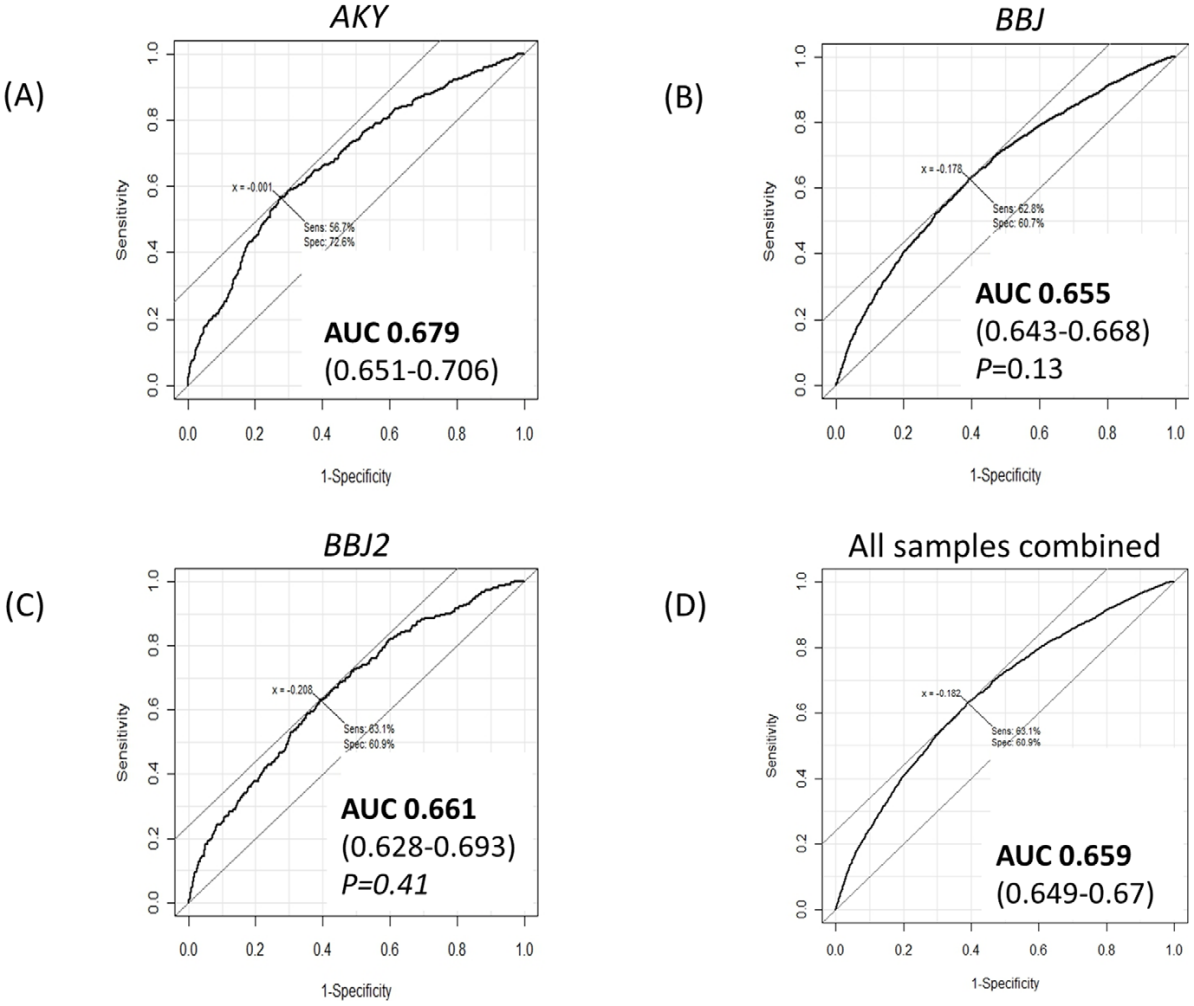
ROC curves for (A) the samples used to create the genetic risk prediction model (*AKY*), (B)(C) the two validation studies (*BBJ* and *BBJ2*), and (D) when all the samples were combined. The AUCs of the model and 95% confidence intervals are indicated. The AUCs of *BBJ* and *BBJ2* are statistically compared with that of *AKY*, and *P*-values are reported. (x: the logarithm of odds ratio at the best cut off, Sens: sensitivity of the model at the best cutoff, Spec: specificity of the model at the best cutoff.)

The AUC and the 95% confidence interval (CI) of the risk prediction model was 0.679 (0.651–0.706) for the model constructing sample set *AKY*. We applied this PC risk prediction model to two other independent Japanese sample sets, *BBJ* and *BBJ2*, and the AUCs were 0.655 (0.643–0.668) for *BBJ*, 0.661 (0.628–0.693) for *BBJ2*, and 0.659 (0.649–0.67) when all the three sample sets were combined (**Figure 1**). There were no statistically significant differences in AUCs between the sample sets, indicating high reproducibility of the predictive performance of this PC risk prediction model in the three independent sample sets. The risk prediction model also showed similar reproducibility (AUC 0.655, 95%CI 0.631–0.679) when it was applied to a sample set of Japanese from the Multiethnic Cohort in Hawaii and California (26) (*MEC*, 980 cases and 1,005 controls) (**Figure S1**).

When we examined the distribution of ORs estimated from the risk prediction model in all the samples used in the present study (*BBJ*, *BBJ2*, and *AKY*), there was almost ten-fold difference in ORs between those in the top and bottom 5 percentile of the population (**Figure S2A**). The ORs of the case samples were modestly but significantly higher than the controls (**Figure S2B**).

### The predictive performance of the genetic risk model is not influenced by serum PSA level

**Figure 2:**
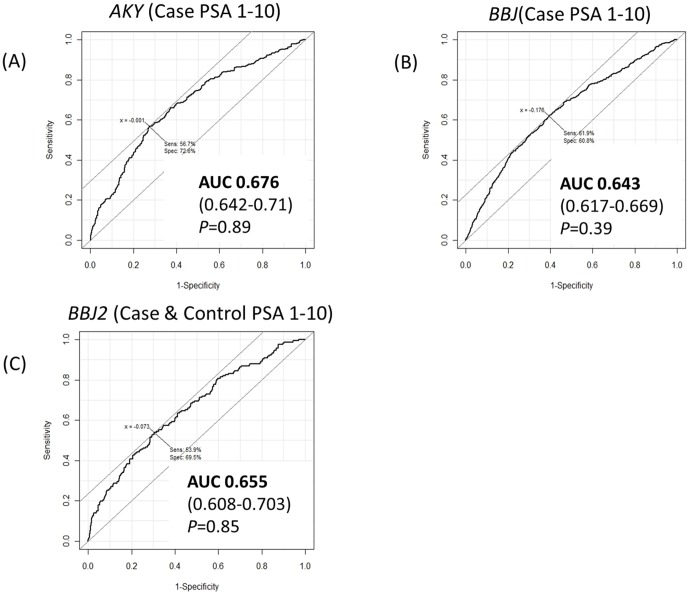
ROC curves of the genetic risk prediction model when the samples were confined to those with PSA 1–10 ng/ml. In (A) *AKY* and (B) *BBJ*, case samples with PSA 1–10 ng/ml and all the control samples are used for analysis. In (C) *BBJ2*, samples are confined to those with PSA 1–10 ng/ml in both case and control samples. The AUCs of the model and 95% confidence intervals are indicated. In each of the sample sets, the AUCs are statistically compared to the AUCs when serum PSA level is not confined (as shown in Figure 1), and *P*-values are reported.

We next evaluated the predictive performance of the model in the samples with serum PSA 1–10 ng/ml, where decisions to proceed to prostate needle biopsies are often difficult to make. Since serum PSA data of the control samples were not available in *AKY* and *BBJ*, the predictive performance was analyzed using the case samples with PSA 1–10 ng/ml and all the controls. In the *BBJ2* nested case-control study samples, serum PSA level was measured for all the case and control samples, and those with PSA 1–10 ng/ml were used for analysis. As a result, AUCs of the risk prediction model were 0.676 (0.642–0.71) for *AKY*, 0.643 (0.617–0.669) for *BBJ*, and 0.655 (0.608–0.703) for *BBJ2* (**Figure 2**). These data were not statistically different from the AUCs observed when the samples were not confined to those with PSA 1–10 ng/ml, indicating that the predictive performance of the risk model is not significantly influenced by serum PSA level. The predictive performance of the risk model was also not influenced by serum PSA level in *MEC* (AUC 0.683, 95% CI 0.631–0.734 for the samples with PSA 1–10 ng/ml) (**Figure S1)**. Furthermore, there was no correlation between PSA and the OR calculated from our risk prediction model (r^2^ = 0.02) (**Figure S3**)

### Application of the genetic prediction model in a clinical scenario

Clinical and practical utilities of biomarkers cannot be assessed only by sensitivity, specificity, or AUC. Prevalence of the disease to be tested or pre-test probability also needs to be considered. To assess the clinical utility of the genetic risk prediction model in individuals with PSA gray-zone, we have calculated the probabilities of positive prostate needle biopsies in genetically high and low risk groups at various cut-off OR values assuming that the overall probability of positive prostate needle biopsies is 20%, a typical value in patients with gray-zone PSA [5], [6], [7]. As a result, there was more than two fold difference in positive prostate biopsy rates between the high and low risk groups at any cutoff OR value (**Table 3**). At the cutoff OR of 0.5, 24.2% of the population was classified as low risk, and the probability of a positive prostate biopsy was 10.7%. On the other hand, at the cutoff OR of 2.0, 9.7% of the population was classified as high risk, and the probability of a positive biopsy was 42.4%. These estimations indicate that although the AUC of the genetic prediction model is not high as a biomarker, it can be clinically and practically useful when it is applied in a limited situation where pre-test probability is relatively high, such as in PSA gray-zone.

**Table 3:** Probability of positive prostate biopsy in high and low risk patients grouped by the genetic risk model at various cutoffs.

Odds ratio cutoff	Percentile of population (%)		Sensitivity (%)	Specificity (%)	Probability of positive biopsy (%)^c^	
	High risk group^a^	Low risk group^b^			High risk group	Low risk group
**0.5**	**75.8**	**24.2**	**85.7**	**29.8**	**23.4**	**10.7**
1	37.9	62.1	52.7	70.6	30.9	14.3
2	9.7	90.3	16.8	94.3	42.4	18.1
3	3.4	96.6	6	98.2	45.5	19.3
5	0.7	99.3	1.2	99.6	42.9	19.9
0.83^d^	47.9	52.1	63.1	60.9	28.7	13.2

a Percentile of population above the cut-off odds ratio in all the samples in the present study.

b Percentile of population below the cutoff odds ratio.

C Probability of positive prostate biopsy assuming the overall positive probability of prostate biopsy to be 20%.

d The best cutoff determined by the ROC analysis.

## Discussions

PSA is a protein secreted specifically from the prostate gland, and has been widely accepted as a serum biomarker for PC. However, other medical conditions, such as benign prostatic hypertrophy and inflammation can cause serum PSA elevation [30]. Hence, the diagnostic specificity of PSA is quite low, especially at boarder-line levels of PSA, or ‘gray-zone’. Patients suspected to have PC by PSA screening usually undergo prostate needle biopsy, which is an invasive procedure that accompany complications, some of which are severe. In addition, recent randomized controlled trials have shown no or little benefit of PSA screening in extending cancer-specific survival [31], [32]. Economic burden of prostate needle biopsies, followed by overdiagnosis and overtreatment for PC, is another serious issue since it is estimated that each year, more than one million patients undergo prostate needle biopsies in the US, a procedure which costs $500–1,000 for each [33], [34]. Therefore, there is a world-wide controversy over PSA screening, and additional biomarkers which can better identify the patients that need prostate needle biopsies are definitely required.

The risk of PC is 2.5 times higher in the patients with a positive family history of PC in their first degree relatives [35]. However, only a small proportion of patients have known positive family history of PC in Asians including the Japanese [36], [37], and collection of detailed family history is often problematic. In our GWAS of the Japanese which includes 4,584 PC, only 6.6% of the patients had positive family history of PC, and data was missing in 15.5% of the patients. Contrarily, risk prediction models based on easily accessible genetic information can be applied to the general population. Furthermore, unlike other biomarkers which show some degree of fluctuation that could affect their reproducibility, genetic risk scores are stable in each individual. So far, few studies have studied the reproducibility of genetic risk prediction models using independent sets of samples. Although reproducibility of genetic risk prediction models could be affected by sample collection bias, using independent sets of samples we have shown that our model is highly reproducible in a single ethnic group. In fact, our model showed similar reproducibility when it was applied to a sample set of Japanese from the Multiethnic Cohort in Hawaii and California (*MEC*). These data warrant similar predictive performance of our model in other Japanese population or East Asian population as well.

Genetic risk prediction of PC was first reported using only five common susceptibility variants [38]. The model was established by simply counting the number of risk alleles. Subsequently, models incorporated increasing number of variants, and logistic regression models were adapted to account for the effect size of each variant [39], [40], [41]. In the present study, we have also created a model based on step-wise logistic regression analysis. When compared to the model incorporating 9 of the 16 variants that remained significant in a step-wise model, the predictive performance of the model including all the 16 variants was modestly but statistically significantly superior (**Figure S4**). Over forty PC susceptibility variants have been reported so far, and still more remains to be identified. Inclusion of novel variants newly identified to be associated with PC is expected to further improve the predictive performance of the model, although careful selection of the variants based on association studies in each ancestry group is necessary. Of note, even when we have included only 16 PC susceptibility variants identified to be associated with PC in GWAS of Japanese, one variant showed negative association with PC in the samples used to create the risk prediction model, suggesting that a variant with very mild effect would show either positive or negative association in different sample sets even in a single ancestry group, which may lower the predictive performance of the model.

There is still a large debate over the clinical utility of genetic risk prediction models. The overall predictive performances of genetic risk prediction models as assessed by ROC analysis are usually modest, since the distribution of the ORs between the case and controls largely overlap. However, it has been implicated in breast cancer that genetic risk prediction models could be clinically useful among a subset of high risk patients [42]. In case of PC, patients can be risk-stratified using PSA, and genetic risk prediction models can be a useful compensatory marker at gray-zone PSA, where patients have relatively high risk of PC, and the diagnostic ability of PSA is the lowest. Furthermore, PCs are generally slow growing, and even if patients with PC are false negatively classified as low risk by a genetic prediction model, they can still be followed with serial PSA measurements, and can have prostate biopsy with increasing PSA before reaching advanced stages except in rare cases of very aggressive tumor. Identification of aggressive PCs is another important issue in PC diagnosis. Most of the PC susceptibility variants identified by GWAS have fallen short of discriminating aggressive from non-aggressive PCs [43], and there was no significant difference in the distribution of ORs between the aggressive and non-aggressive PCs in our genetic risk prediction model as well (**Figure S5**). Additional biomarkers that can discriminate aggressive and indolent PCs should be explored.

The circumstance where biomarkers are applied is also important in assessing their clinical values. When the prevalence of a disease is very low, even a marker with very high sensitivity and specificity shows low positive predictive value. Since the overall predictive value of genetic risk prediction models are low, it is important to utilize the model in a situation where disease prevalence or pre-test probability is relatively high. We have demonstrated that the predictive performance of the genetic risk prediction model is not affected by serum PSA level, and that the model can be utilized in the patients with gray-zone PSA, where there is usually 20–25% probability of a positive prostate needle biopsy. Recently, a few common variants have been reported to be associated with serum PSA level [44], and two of them, rs10993994 at *10q11* and rs7501939 at *17q12* were included in our genetic risk prediction model. It is presumed that the small effect size of these variants on PSA level did not significantly affect overall predictive performance of our risk prediction model at gray-zone PSA. Some other studies that have evaluated a model incorporating PSA and genetics have argued that there was only minor increase in AUC when genetic information was added [45]. However, PSA fluctuation is problematic in PSA gray-zone, and it is possible that the true effect of combining PSA with genetic risk information is confounded by these variances in PSA.

We have shown that while the genetic risk model may not be helpful clinically in all the patients with gray-zone PSA, it may largely influence decision making in a portion of patients. In our clinical simulation, 24.2% of the patients had OR<0.5, and these patients had 10.7% chance of being positive after prostate needle biopsy. Considering the complications of prostate needle biopsies, these patients might chose serial PSA follow-up rather than immediate prostate needle biopsy. On the other hand, 9.7% of the patients with OR>2, who have more than 42.4% chance of being positive for prostate cancer, may choose to undergo immediate prostate needle biopsy. Although the genetic risk prediction model should further be evaluated prospectively in clinics, our data suggests that it can be an additional biomarker that can risk-stratify individuals at gray-zone PSA in Japanese, leading to personalized medicine.

## Conclusions

We have created a genetic risk prediction model based on 16 common variants shown to be associated with PC in Japanese GWAS. The model was highly reproducible and its predictive performance was not affected by PSA. The model has a potential to be a clinically useful biomarker that can risk stratify patients at gray-zone PSA, which should be confirmed in future prospective studies.

## Supporting Information

Table S1 **Summary results of the previously reported SNPs associated with prostate cancer susceptibility in GWAS of the Japanese.**
(DOCX)

Table S2 **The results of association study for rs620861.**
(DOCX)

Figure S1 **Reproducibility of the model in** ***MEC*** **Japanese samples.** ROC curves of the risk prediction model in a set of Japanese samples from the Multiethnic Cohort in Hawaii and California (*MEC*). Right panel shows the ROC curve when all the samples were used. Left panel shows the ROC curve for the samples with PSA 1–10 ng/ml (only the samples whose serum PSA levels were known before genotyping are included). The AUCs of the model and 95% confidence intervals are indicated.
(TIF)

Figure S2 **Distribution of odds ratio estimated from the prediction model.** (A) Distribution of odds ratio in all the case and control samples used for analysis in the present study. Odds ratio at each percentile of population is described. (B) Distribution of odds ratio in case (red) and control (blue) samples are plotted. The difference in odds ratio between case and controls are statistically compared by Student's *t*-test, and the *P*-value is reported.
(TIF)

Figure S3 **Correction between serum PSA level and OR.** Serum PSA level and OR are plotted for all the case and control samples with PSA 1–10 ng/ml, and the correlation coefficient (R^2^) is indicated.
(TIF)

Figure S4 **A risk prediction model created by step-wise logistic regression.** ROC analysis of a risk prediction model created by step-wise logistic regression. 9 variants that remained significant were incorporated into the model. The ROC plot is based on the combined data of all three sample sets used in the present study. The AUC of the model and 95% confidence intervals are indicated. The AUC of the model is statistically compared with that of the model created by unconditional logistic regression based on 16 SNPs by DeLong's test, and the *P*-value is reported.
(TIF)

Figure S5 **The distribution of odds ratio in high and non-high risk tumors.** Distribution of odds ratio in *AKY* are plotted separately in high (red) and non-high (blue) risk tumors. High risk tumors fulfill either of the following criteria; PSA≥20, or GS≥8, or clinical stage≥C. Odds ratio between high and non-high risk tumors are statistically compared by Student's *t*-test, and the *P*-value is reported. *Y*-axis shows cumulative frequency.
(TIF)
