# Supplementary material for: Reproducibility, Performance, and Clinical Utility of a Genetic Risk Prediction Model for Prostate Cancer in Japanese
Source: PLoS One. 2012 Oct 10;7(10):e46454. doi: 10.1371/journal.pone.0046454 (PMC3468627; doi:10.1371/journal.pone.0046454)
Supplement: Table S1 — Summary results of the previously reported SNPs associated with prostate cancer susceptibility in GWAS of the Japanese. (DOCX) [file pone.0046454.s001.docx]

| **Table S1 Summary results of the previously reported SNPs　associated with prostate cancer susceptibility in GWAS of the Japanese** | | | | | | | |
| --- | --- | --- | --- | --- | --- | --- | --- |
|  |  |  |  |  |  |  |  |
|  |  |  |  |  |  |  |  |
| **Chr** | **Region** |  | **Reported SNP** |  | **GWAS in Japanese population** | | |
|  |  |  | **refSNP ID** |  | **refSNP ID ^a^** | **Odds ratio** | ***P* value ^b^** |
|  |  |  |  |  |  |  |  |
| 2 | EHBP1 |  | rs721048 |  | rs2710646^f^ | 1.04 | 6.8 x 10^-01^ |
| 2 | THADA |  | rs1465618 |  | rs1465618 | 1.23 | **1.0 x 10^-05^** |
| 2 | ITGA6 |  | rs12621278 |  | rs12621278 | 1.11 | 5.7 x 10^-02^ |
| 3 | 3p12 |  | rs2660753 |  | rs2660753 | 1.23 | **1.3 x 10^-05^** |
| 3 | 3q21 |  | rs10934853 |  | rs10934853 | 1.06 | 1.6 x 10^-01^ |
| 4 | PDLIM5 |  | rs17021918 |  | rs17021918 | 1.05 | 3.1 x 10^-01^ |
| 4 | TET2 |  | rs7679673 |  | rs7679673 | 1.13 | 2.2 x 10^-02^ |
| 6 | SLC22A3 |  | rs9364554 |  | rs9364554 | 1.03 | 4.8 x 10^-01^ |
| 7 | LMTK2 |  | rs6465657 |  | rs6465657 | 1.02 | 7.3 x 10^-01^ |
| 8 | NKX3-1 |  | rs1512268 |  | rs1512268 | 1.34 | **4.3 x 10^-11^** |
| 8 | 8q24 (Block1) | | rs12543663 |  | rs12543663 | 1.07 | 4.2 x 10^-01^ |
| 8 | 8q24 (Block1) | | rs10086908 |  | rs10086908 | 1.28 | **6.9 x 10^-06^** |
| 8 | 8q24 (Block2 / Region2) | | rs1016343 |  | rs1016343 | 1.39 | **2.7 x 10^-13^** |
| 8 | 8q24 (Block2 / Region2) | | rs13252298 |  | rs13252298 | 1.40 | **6.7 x 10^-13^** |
| 8 | 8q24 (Block2 / Region2) | | rs6983561 |  | rs16901966 | 1.44 | **1.9 x 10^-12^** |
| 8 | 8q24 (Block3 / Region3) | | rs16902094 |  | rs16902094 | 1.13 | 1.9 x 10^-02^ |
| 8 | 8q24 (Block3 / Region3) | | rs445114 |  | rs445114 | 1.15 | **9.3 x 10^-04^** |
| 8 | 8q24 (Block3 / Region3) | | rs620861 |  | rs620861 | 1.16 | **7.2 x 10^-04^** |
| 8 | 8q24 (Block4 / Region3) | | rs6983267 |  | rs6983267 | 1.23 | **2.9 x 10^-06^** |
| 8 | 8q24 (Block5 / Region1) | | rs1447295 |  | rs1447295 | 1.61 | **6.0 x 10^-20^** |
| 8 | 8q24 (Block5 / Region1) | | rs10090154 |  | rs7837688^f^ | 1.75 | **1.2 x 10^-25^** |
| 10 | MSMB |  | rs10993994 |  | rs10993994 | 1.27 | **3.4 x 10^-08^** |
| 10 | CTBP2 |  | rs4962416 |  | rs4962416 | 0.99 | 9.7 x 10^-01^ |
| 11 | 11p15 |  | rs7127900 |  | rs7127900 | 1.01 | 4.3 x 10^-01^ |
| 11 | 11q13 |  | rs7931342 |  | rs7931342 | 1.07 | 1.6 x 10^-01^ |
| 17 | HNF1B |  | rs4430796 |  | rs4430796 | 1.30 | **1.2 x 10^-08^** |
| 17 | 17q24 |  | rs1859962 |  | rs1859962 | 0.99 | 8.4 x 10^-01^ |
| 19 | 19q13 |  | rs8102476 |  | rs8102476 | 1.01 | 7.7 x 10^-01^ |
| 19 | KLK2/KLK3 |  | rs2735839 |  | rs2735839 | 1.14 | 3.3 x 10^-03^ |
| 22 | TTLL1/BIK |  | rs5759167 |  | rs5759167 | 1.17 | **5.8 x 10^-04^** |
| X | NUDT10/NUDT11 |  | rs5945619 |  | rs5945619 | 1.38 | 8.6 x 10^-03^ |
| This table lists the PC-susceptibility loci reported by July, 2010. The SNPs incorporated in the genetic risk prediction model are underlined.　^a^ When the exact reported SNP was not studied in the GWAS of the Japanese, a SNP indicating strong LD (r^2^ ≥ 0.8) with the reported SNP in Phase II of HapMap CEU data was used as an alternative. ^b^ The 15 SNPs that passed the significance level after Bonferroni correction (0.05/31= 1.6x10^-3^) are indicated in bold letters. | | | | | | | |
|  |  |  |  |  |  |  |  |
