# Supplementary material for: Reproducibility, Performance, and Clinical Utility of a Genetic Risk Prediction Model for Prostate Cancer in Japanese
Source: PLoS One. 2012 Oct 10;7(10):e46454. doi: 10.1371/journal.pone.0046454 (PMC3468627; doi:10.1371/journal.pone.0046454)
Supplement: Table S2 — The results of association study for rs620861. (DOCX) [file pone.0046454.s002.docx]

| **Table S2 The results of association study for rs620861** | | | | | | | |  |  |  |  |  |
| --- | --- | --- | --- | --- | --- | --- | --- | --- | --- | --- | --- | --- |
|  | case | | |  | control | | | | Trend *P* ^a^ | OR ^b^ | 95% CI | *P*het ^c^ |
| Number of risk allele | 0 | 1 | 2 |  | 0 | 1 | 2 | |  |  |  |  |
| *AKY* | 135 | 348 | 206 |  | 143 | 361 | 245 | | 0.37 | 0.93 | (0.81-1.08) |  |
| *BBJ1* | 603 | 1447 | 900 |  | 1136 | 2585 | 1515 | | 0.08 | 1.06 | (0.99-1.13) |  |
| *BBJ2* | 66 | 171 | 107 |  | 215 | 499 | 331 | | 0.85 | 1.02 | (0.85-1.21) |  |
| Combined | 804 | 1966 | 1213 |  | 1494 | 3445 | 2091 | | 0.22 | 1.04 | (0.98-1.10) | 0.31 |
| ^a^ *P* for trend (1-degree of freedom) ^b^ Odds ratio and confidence intervals of the risk allele in multiplicative models | | | | | | | | | | | |  |
| ^c^ The Breslow-Day test for heterogeneity | | |  |  |  |  |  | |  |  |  |  |
