# Supplementary figures and images for: Reproducibility, Performance, and Clinical Utility of a Genetic Risk Prediction Model for Prostate Cancer in Japanese
Source: PLoS One. 2012 Oct 10;7(10):e46454. doi: 10.1371/journal.pone.0046454 (PMC3468627; doi:10.1371/journal.pone.0046454)

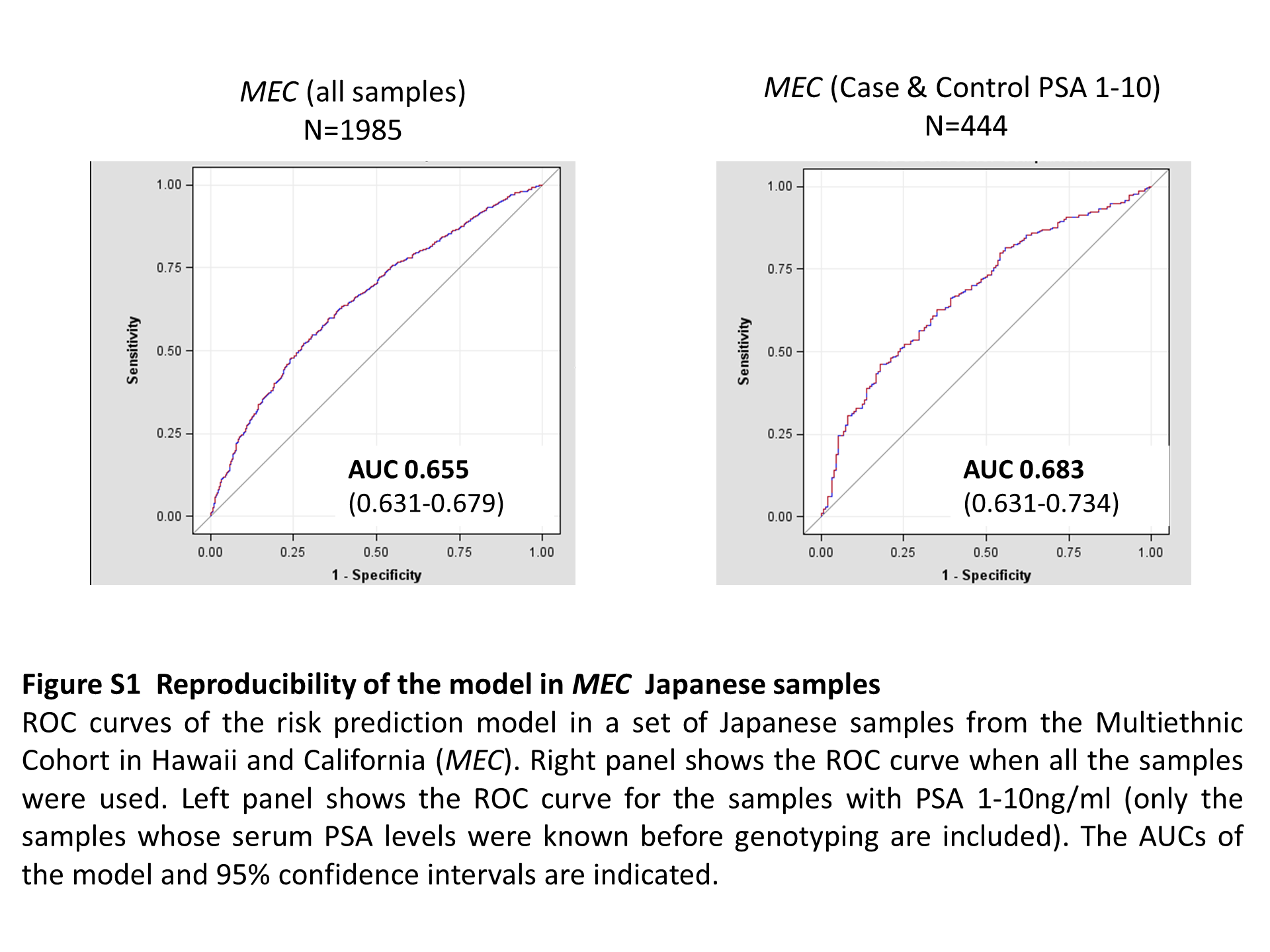

Supplement: Figure S1 — Reproducibility of the model in MEC Japanese samples. ROC curves of the risk prediction model in a set of Japanese samples from the Multiethnic Cohort in Hawaii and California (MEC). Right panel shows the ROC curve when all the samples were used. Left panel shows the ROC curve for the samples with PSA 1–10 ng/ml (only the samples whose serum PSA levels were known before genotyping are included). The AUCs of the model and 95% confidence intervals are indicated. (TIF) [file pone.0046454.s003.tif]

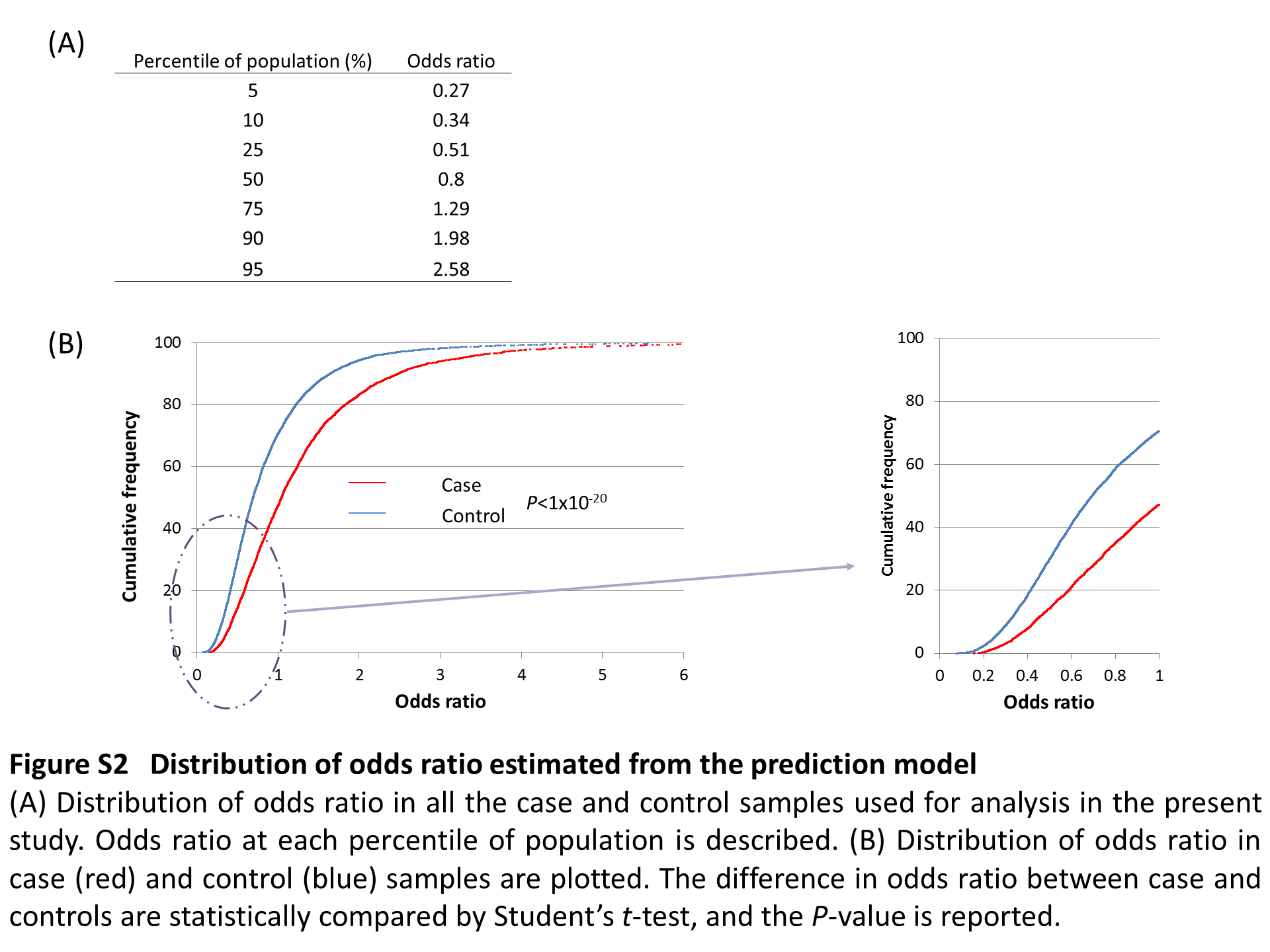

Supplement: Figure S2 — Distribution of odds ratio estimated from the prediction model. (A) Distribution of odds ratio in all the case and control samples used for analysis in the present study. Odds ratio at each percentile of population is described. (B) Distribution of odds ratio in case (red) and control (blue) samples are plotted. The difference in odds ratio between case and controls are statistically compared by Student's t-test, and the P-value is reported. (TIF) [file pone.0046454.s004.tif]

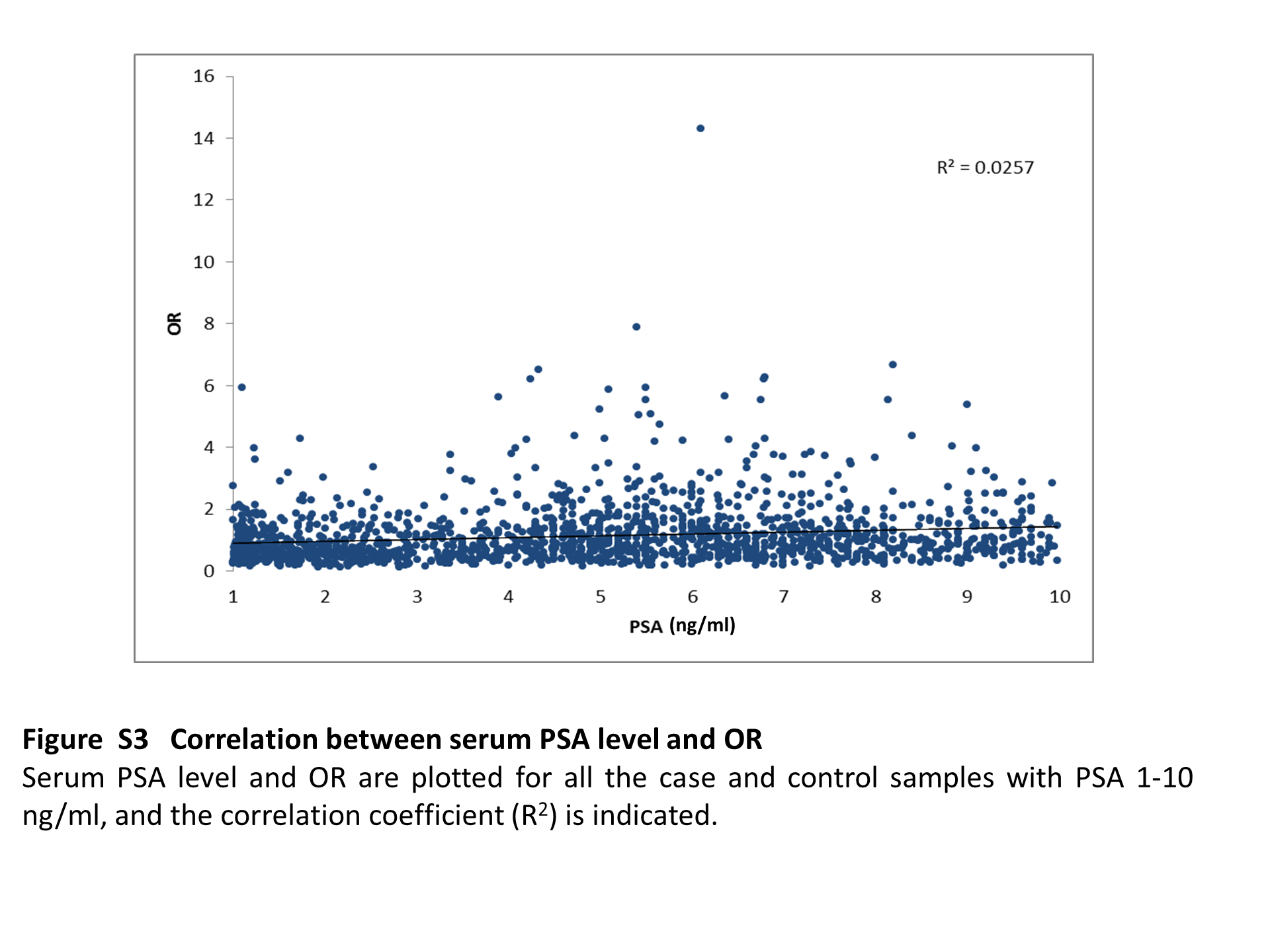

Supplement: Figure S3 — Correction between serum PSA level and OR. Serum PSA level and OR are plotted for all the case and control samples with PSA 1–10 ng/ml, and the correlation coefficient (R2) is indicated. (TIF) [file pone.0046454.s005.tif]

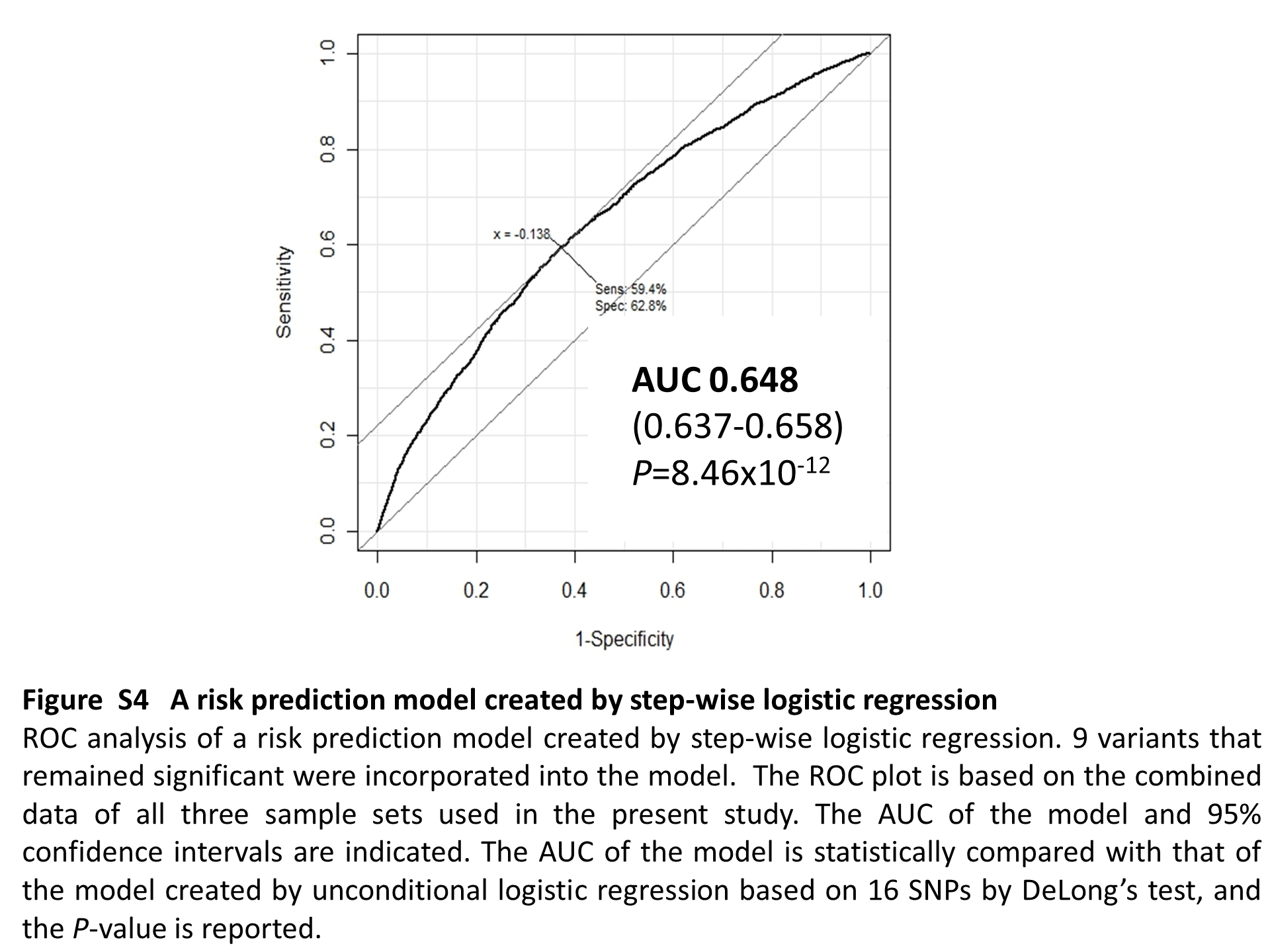

Supplement: Figure S4 — A risk prediction model created by step-wise logistic regression. ROC analysis of a risk prediction model created by step-wise logistic regression. 9 variants that remained significant were incorporated into the model. The ROC plot is based on the combined data of all three sample sets used in the present study. The AUC of the model and 95% confidence intervals are indicated. The AUC of the model is statistically compared with that of the model created by unconditional logistic regression based on 16 SNPs by DeLong's test, and the P-value is reported. (TIF) [file pone.0046454.s006.tif]

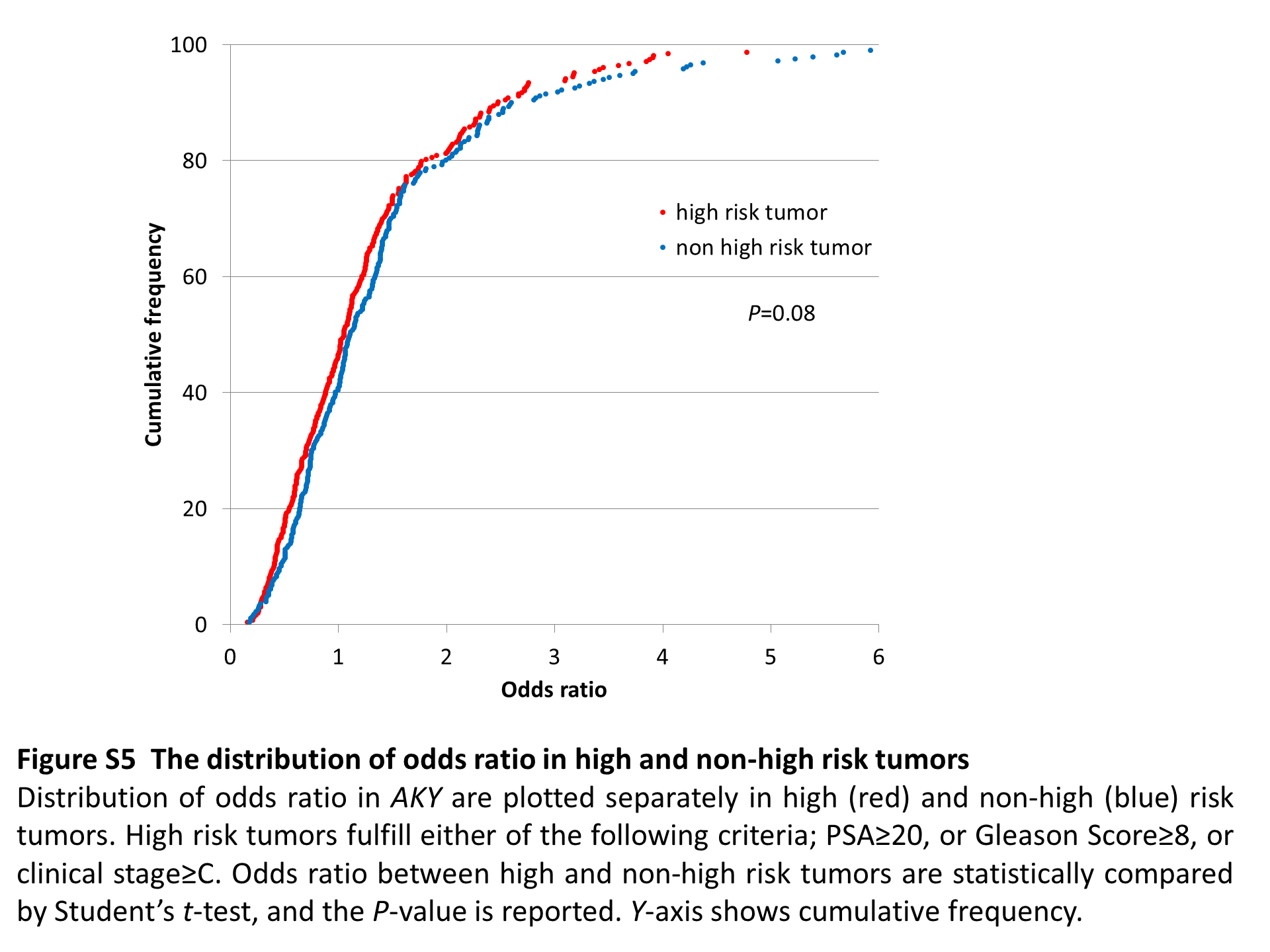

Supplement: Figure S5 — The distribution of odds ratio in high and non-high risk tumors. Distribution of odds ratio in AKY are plotted separately in high (red) and non-high (blue) risk tumors. High risk tumors fulfill either of the following criteria; PSA≥20, or GS≥8, or clinical stage≥C. Odds ratio between high and non-high risk tumors are statistically compared by Student's t-test, and the P-value is reported. Y-axis shows cumulative frequency. (TIF) [file pone.0046454.s007.tif]
